# The Rev. Dr. Anson Judd Upson

**Published:** 1902-07

**Authors:** 


THE Rev. Dr. Anson Judd Upson, chancellor of the University of the State of New York and ex-president of Auburn Theological Seminary, died at his home at Glens Falls, at the age of 82. Chancellor Upson was a pronounced friend of higher educational standards, having been engaged during all of his active life either in teaching or in some way promoting advancements in educational methods. In 1874 he was elected by the legislature a regent of the University of the State of New York; he was made vice-chancellor in 1890, and in 1892 was elected chancellor of the University as the successor of George William Curtis. In his capacity as chancellor he had signed every state license to practise medicine since the law creating state examining boards went into effect.

